# Genomic Characterization and Resistance Mechanisms of Carbapenem-Resistant *Klebsiella pneumoniae* ST101 Isolates from Saudi Arabia

**DOI:** 10.3390/ijms262311518

**Published:** 2025-11-27

**Authors:** Enaam K. Idrees, Manal M. Alkhulaifi, Marwh G. Aldriwesh, Nasser Alqurainy, Liliane Okdah, Abdulrahman A. Alswaji, Eisa T. Alrashidi, Alhanouf S. Alshahrani, Sameera M. Al Johani, MNGHA Surveillance Group, Hanan H. Balkhy, Majed F. Alghoribi

**Affiliations:** 1 Department of Botany and Microbiology, College of Science, King Saud University, Riyadh 11451, Saudi Arabia; enaam.khalid@gmail.com (E.K.I.);; 2 Infectious Diseases Research Department, King Abdullah International Medical Research Center, Riyadh 11481, Saudi Arabia; aldriweshm@ksau-hs.edu.sa (M.G.A.);; 3 Department of Clinical Laboratory Sciences, College of Applied Medical Sciences, King Saud bin Abdulaziz University for Health Sciences, Riyadh 11481, Saudi Arabia; 4 Ministry of the National Guard-Health Affairs, Riyadh 11426, Saudi Arabia; info@mngha.med.sa; 5 Department of Basic Science, College of Science and Health Professions, King Saud bin Abdulaziz University for Health Sciences, Riyadh 14611, Saudi Arabia

**Keywords:** *Klebsiella pneumoniae*, ST101, carbapenem resistance, *bla*_OXA-48_, *bla*_NDM-1_, hypervirulent CRKP, whole-genome sequencing, plasmid-mediated resistance

## Abstract

Carbapenem-resistant *Klebsiella pneumoniae* (CRKP) represents a critical global health threat, with ST101 identified as a major circulating clone in Saudi Arabia. We used whole genome sequencing and plasmid reconstruction to investigate the molecular characteristics of CRKP ST101 isolates from Saudi Arabia (2018–2021), analyzing antimicrobial resistance genes (ARGs), virulence factors, and plasmid structure and replicon types. Clinical isolates were obtained from the Ministry of National Guard Health Affairs (MNGHA) hospitals in Saudi Arabia between 2018 and 2021. Whole-genome sequencing was performed using the Illumina MiSeq^®^ platform, followed by comprehensive bioinformatic analysis of ARGs, virulence factors, and plasmid content. All ten isolates belonged to ST101 and harbored extensive antimicrobial resistance (AMR) and virulence determinants. Nine isolates (90%) carried *bla*_OXA-48_, with three co-harboring *bla*_NDM-1_, representing dual-carbapenemase producers. These carbapenemase genes were located on plasmids with distinct replicon types, including IncL/M, IncHI1B/IncFIB, and IncFIA/IncR. All isolates were multidrug-resistant (MDR), with half classified as extensively drug-resistant (XDR). Four isolates exhibited hypervirulent profiles, harboring aerobactin and yersiniabactin siderophores. This study provides comprehensive genomic characterization of CRKP ST101 in Saudi Arabia, revealing complex resistance mechanisms mediated by diverse plasmid types. The findings highlight the importance of genomic surveillance to track the evolution and dissemination of high-risk MDR and XDR lineages and inform targeted infection control strategies.

## 1. Introduction

*Klebsiella pneumoniae* is a leading causative agent of a wide variety of community-acquired and hospital-acquired infections, including liver abscess, pneumonia, bloodstream infections, and urinary tract infections [1]. The growing acquisition of ARGs has significantly reduced the effectiveness of available treatment options, including those involving last-resort antibiotics [2,3,4]. In response to this escalating threat, the World Health Organization (WHO) included *K. pneumoniae* in the highest priority tier of its 2024 Bacterial Priority Pathogens List (BPPL) to guide research, policy efforts and strategies aimed at combating AMR [5]. The rapid emergence and dissemination of CRKP strains represent a significant public health concern and economic burden worldwide [6,7,8]. Multiple molecular mechanisms contribute to CRKP, often complicating therapeutic management. Key resistance mechanisms include the overproduction of β-lactamases combined with reduced outer membrane permeability, as well as the acquisition of one or more carbapenemase enzymes capable of hydrolyzing carbapenem antibiotics [9,10]. According to the Ambler classification, the major classes of carbapenemases include class A *Klebsiella pneumoniae* carbapenemase (KPC), class B Metallo-β-lactamases such as Verona Integron-encoded metallo-β-lactamase (VIM), imipenemase (IMP), and New Delhi metallo-β-lactamase (NDM), and class D (OXA-48-like oxacillinases) [11,12,13]. First identified in 2001, *bla*_OXA-48_ has become the most frequently reported carbapenemase gene in *Enterobacterales* and is strongly associated with hospital outbreaks worldwide [13,14,15,16,17,18]. Among the high-risk clones, sequence type 101 (ST101) has been particularly associated with the dissemination *bla*_OXA-48_ in diverse clinical settings [19]. *K. pneumoniae* ST101 was first defined in 2008 during the development of the multilocus sequence typing (MLST) scheme for categorizing *K. pneumoniae,* although details regarding its initial clinical emergence remain limited in the published literature. Over the past decade, ST101 has gained recognition as a high-risk clone associated with AMR, supported by its increasing detection in clinical and environmental isolates worldwide [20,21]. Notable cases of CRKP ST101 strains emerged in Italy and other European countries during the early 2010s, prompting public health concerns due to their resistance to critical antibiotics [22,23]. In Saudi Arabia, ST101 has been identified as one of the predominant *K. pneumoniae* clones implicated in the regional dissemination of CRKP strains [24]. Despite its recognized clinical importance, comprehensive genomic data on ST101 in Saudi Arabia remain limited. This lack of molecular insight hinders our ability to understand its resistance mechanisms, virulence potential, and patterns of regional transmission. In recent years, whole genome sequencing has become a critical tool for elucidating the transmission dynamics and genetic determinants underlying the persistence and dissemination of high-risk *K. pneumoniae* clones. In response to this knowledge gap, we performed molecular characterization of CRKP ST101 isolates from Saudi Arabia, focusing on sequence typing, ARGs, and key virulence determinants collected over a four-year period (2018–2021). This study represents the first comprehensive genomic analysis of CRKP ST101 isolates in Saudi Arabia, offering novel insights into the distribution of resistance and virulence determinants alongside detailed plasmid architecture, and deepening our understanding of this high-risk clone’s emergence and potential for regional spread.

## 2. Results

### 2.1. Clinical and Geographic Distribution of ST101 Isolates

**Figure 1:**
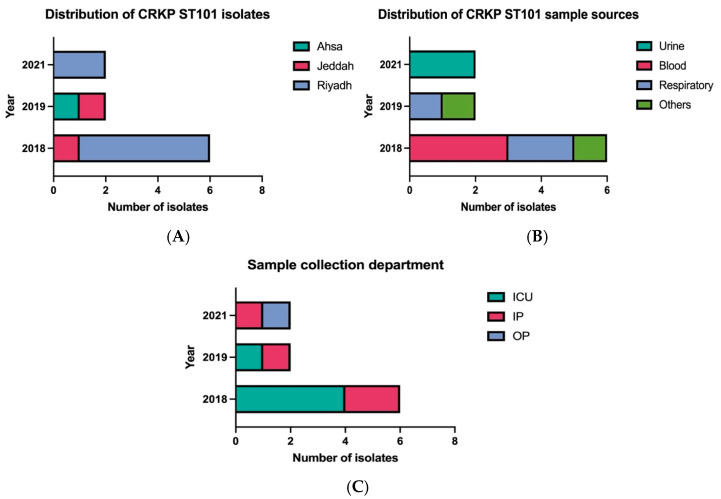
Epidemiological characteristics of CRKP ST101 isolates. (**A**) Geographic distribution across Ahsa, Jeddah, and Riyadh (from 2018 to 2021). (**B**) Distribution by specimen type, including blood, respiratory samples, urine, and wound or rectal swabs. (**C**) Distribution by clinical setting (intensive care unit, inpatient ward, or outpatient clinic) over the study period.

Ten CRKP clinical isolates were obtained from MNGHA hospitals in Saudi Arabia between January 2018 and December 2021, representing 4.5% of all CRKP isolates identified through the institutional AMR surveillance program. Geographic distribution showed Riyadh as the primary source (n = 7), followed by Jeddah (n = 2), and Ahsa (n = 1) (Figure 1A). The isolates originated from a range of clinical specimens, with blood and respiratory samples being the most frequent (n = 3, each), followed by urine (n = 2), and swabs from wound and rectal sites (n = 1, each) (Figure 1B). A temporal analysis revealed that the majority of isolates (n = 6) were recovered in 2018, followed by a sharp decline in 2019 and 2020 (n = 2, each year). No isolates were identified in 2021, likely due to COVID-19-related restrictions affecting surveillance activities. Nine of the ten patients were hospitalized, with a single case identified in an outpatient setting. Among the hospitalized patients, five were located in intensive care units and four were in internal medicine wards. All patients were Saudi nationals with no documented history of international travel (Figure 1C).

### 2.2. Clinical Characteristics and Epidemiology of CRKP ST101 Isolates

Clinical data from patients in Riyadh and Jeddah were retrieved from the hospitals’ electronic medical records systems in compliance with ethical approval guidelines. Clinical data of one patient in Ahsa were not available. The patients’ ages ranged from 38 and 92 years. Primary admission diagnoses included respiratory infections and respiratory distress (n = 2), with other presentations detailed in Table 1. Six patients (67%) developed healthcare-associated infections during hospitalization. CRKP isolation occurred ≥48 h after admission in these cases, consistent with nosocomial acquisition. Clinical outcomes were severe. Six patients (67%) developed sepsis that progressed to septic shock and multiorgan failure, and as a result all six died during hospitalization. Common comorbidities included diabetes mellitus and hypertension, with complete patient profiles provided in Table 1.

### 2.3. Phenotypic Antimicrobial Resistance Profiles

Bacterial identification and antimicrobial susceptibility testing were performed using the VITEK II automated system with the AST-N291 card (bioMérieux, Marcy-l’Étoile, France) according to Clinical and Laboratory Standards Institute (CLSI) guidelines [25]. All isolates demonstrated resistance to multiple β-lactam antibiotics, including penicillins (ampicillin, amoxicillin/clavulanic acid, piperacillin/tazobactam) and cephalosporins (cefaclor, cefoxitin, ceftazidime, ceftriaxone, cefepime), as well as fluoroquinolones (ciprofloxacin) and other agents (nitrofurantoin, and trimethoprim). High but variable resistance was observed for meropenem (n = 9), amikacin (n = 7), gentamicin (n = 7), and trimethoprim/sulfamethoxazole (n = 7). Tigecycline susceptibility was retained in all isolates, representing the only universally effective agent tested. All isolates were classified as MDR, defined as resistance to agents in three or more antimicrobial categories. Five isolates (50%) were classified as XDR, with susceptibility limited to tigecycline and one or two additional agents.

### 2.4. Genomic Analysis of the ST101 Isolate

All isolates were identified as sequence type (ST) 101 by multilocus sequence typing (MLST), with an average genome size of 5,851,131 bp and a GC content of 56.73%. Assembly statistics revealed an average of 191 contigs per genome, with the largest contig measuring 5,598,060 bp and an average N50 value of 2,362,430 bp. Serotyping revealed a uniform capsular profile across all isolates, with K-locus KL17, O-locus O1/O2v1, and wzi-type wzi137. Plasmid content ranged from 2 to 9 per isolate, with an average of 5. Kleborate resistance scoring revealed high resistance levels: five isolates (50%) achieved the maximum score of 3 (carbapenemase plus colistin resistance), four (40%) scored 2 (carbapenemase only), and one (10%) scored 1 (ESBL only). Kleborate virulence scoring identified two predominant profiles: six isolates (60%) carried yersiniabactin only (score 1), while four (40%) harbored both aerobactin and yersiniabactin (score 4).

### 2.5. Antimicrobial Resistance Mechanisms

The CRKP clinical isolates exhibited Kleborate resistance scores of 1–3, harboring 6 to 15 resistance genes conferring resistance to 6–10 antimicrobial classes. The isolates demonstrated resistance to most clinically relevant antimicrobials through diverse resistance mechanisms. β-lactamase genes, including *bla*_OXA-9_ and *bla*_TEM-1_, were identified in seven (n = 7). Three *bla*_CTX-M_ Extended-spectrum β-lactamases (ESBL) variants were identified, *bla*_CTX-M-14_ (n = 1), *bla*_CTX-M-15_ (n = 7), and *bla*_CTX-M-27_ (n = 4), with *bla*_CTX-M-15_ being most prevalent. Nine isolates (90%) harbored carbapenemase genes, including *bla*_OXA-48_ (n = 9) being most prevalent and *bla*_NDM-1_ present in three isolates (30%). Three isolates (30%) co-harbored both carbapenemases, representing dual-carbapenemase producers with enhanced resistance potential. All isolates harbored aminoglycoside resistance genes, including *aph*(3′), *armA*, *aadA*, *aac*(6′), and *sat*-2. One isolate harbored the fluoroquinolone resistance gene *qnrS*, while all the isolates harbored the mutated *gyr*A, and *par*C chromosomal genes that confer resistance against fluoroquinolone in all isolates. Additionally, six isolates harbored one or two of sulfonamides resistance genes *sul*1 and/or *sul*2, and one isolate harbored the mutated *tet*(D) gene conferring resistance to tetracyclines. All isolates harbored genes conferring resistance to trimethoprim, such as *dfr*A5, *dfr*A14, and *dfr*A1. No plasmid-mediated colistin resistance genes were detected; however, chromosomal mutations in *mgrB* associated with colistin resistance were identified in five isolates (50%).

### 2.6. Virulence Associated Genes

The CRKP isolates exhibited Kleborate virulence scores of 1 and 4, indicating variable virulence potential. Four isolates were classified as hypervirulent CRKP, harboring virulence genes *iuc*ABCD and *iut*A (virulence score 4). All isolates harbored the iron-scavenging siderophore yersiniabactin (*ybt*9), located on the integrative conjugative element ICEKp3. The presence of *ybt*9/ICEKp3 combination in *K. pneumoniae* enhances pathogenicity by linking iron-scavenging capability with resistance mechanisms, contributing to treatment challenges. Aside from plasmid-driven resistance, the detection of ICEKp3 in all isolates reinforces the role of integrative and conjugative elements as conserved genomic features in *K. pneumoniae* ST101 that could increase its virulence potential. The current analysis examined resistance derived from plasmids; however, the presence of ICEKp variants could also contribute to horizontal gene transfer and genomic diversification. Future long-read sequencing approaches will enable comprehensive mapping and structural validation of these elements. Importantly, four isolates harbored virulence regulators (*rmp*A/*rmp*A2) and the siderophore aerobactin (*iuc*), confirming hypervirulent classification. No colibactin was identified in the isolates and complete virulence gene profiles are detailed in (Table 2).

### 2.7. Plasmid Characterization and Resistance Gene Environments

Comprehensive plasmid profiling revealed that all isolates harbored multiple plasmids, ranging from 2 to 9 plasmids per isolate (average: 5 plasmids). Replicon typing using the PlasmidFinder database implemented in ABRicate identified diverse plasmid types, including colicinogenic plasmids (Col440, ColKP3, ColpVC, and ColRNA) and incompatibility groups (IncFIA, IncFIB, IncFII, IncHI1B, IncL/M, and IncR). The most prevalent replicon type was IncL/M, detected in 9/10 isolates (90%), followed by IncFIB and Col440 in 7/10 isolates (70% each). IncHI1B was identified in 6/10 isolates (60%), while ColRNA and IncR were present in 4/10 isolates (40% each). Less frequent replicon types included IncFIA in 2/10 isolates (20%) and IncFII in 1/10 isolates (10%).

**Figure 2:**
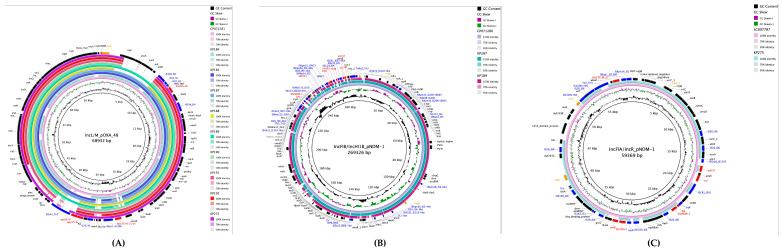
Circular comparison of carbapenemase-carrying plasmids with related reference plasmids. (**A**) IncL/M plasmid harboring *bla*_OXA-48_ (68,932 bp) compared with related plasmids from the literature. (**B**) IncHI1B/IncFIB hybrid plasmid harboring *bla*_NDM-1_ (269,326 bp) compared with related reference plasmids. (**C**) IncFIA/IncR hybrid plasmid harboring dual *bla*_NDM-1_ genes (59,369 bp) compared with related reference plasmids. ARGs are highlighted in red, insertion sequences in blue, and other genetic elements are color-coded according to the legend.

The IncL/M replicon harboring *bla*_OXA-48_ was identified in 9/10 isolates (90%) and measured 68,932 bp in length. BLAST (version 2.17.0) comparison demonstrated 100% query coverage and identity with the reference *bla*_OXA-48_ bearing plasmid IncL/M(pOXA-48)_1_pOXA-48 (Accession number: JN626286). The *bla*_OXA-48_ gene was flanked by insertion sequences IS1D and IS10A, with the complete genetic environment consisting of IS10A-IS1R-IS1D-*bla*_OXA-48_-dmIR-IS10A. An additional ESBL gene (*bla*_CTX_M-15_) was located on the same plasmid and flanked by the ISEcp1 insertion sequence, demonstrating the multi-resistance capacity of this IncL/M plasmid (Figure 2A).

A hybrid plasmid combining IncHI1B and IncFIB replicons was identified harboring *bla*_NDM-1_, with a total length of 269,326 bp. This plasmid carried multiple ARGs, including *aphA*, *msrE*, *rmtB*, and ant, in addition to the carbapenemase gene. The *bla*_NDM-1_ gene was flanked by ISAba125 and ISEc33 insertion sequences, embedded within a complex genetic environment consisting of IS4321R-ISStma11-Tn2-IS26-*hin*-ISAba125-*groS*-*trpF*-*bla*_NDM-1_-ISEc33-ISAba125-*aphA*-ISAba14-IS26-ISKpn26-ISKpn21-ISBcen27-*msrE*-ISEc29-*rmtB*-ISEc35-folP-*emrE*-*ant*-ISSsu9-*xerC*-*hin*-TnAs2-IS4321L. This dense clustering of ARGs within multiple mobile genetic elements characterized the complex architecture of the IncHI1B/IncFIB hybrid plasmid (Figure 2B).

A second hybrid plasmid combining IncFIA and IncR replicons was identified harboring two copies of *bla*_NDM-1_ alongside *bla*_CTX-M-27_, with a total length of 59,369 bp. BLAST comparison showed 83% query coverage and identity with a reference *bla*_NDM-1_-bearing plasmid (Accession number: LC807787). The genetic environment of the *bla*_NDM-1_ genes on this plasmid differed from that observed on the IncHI1B/IncFIB plasmid, with flanking sequences IS26 and ISCR1 and a genetic environment consisting of IS26- *bla*_NDM-1_-*ble*-*trpF*-*dsbD*-ISCR1. The co-located *bla*_NDM-1_ gene was flanked by IS26 and ISEcp1, with a genetic environment of IS26-Tn2-*wbuC*- *bla*_CTX-M-27_-ISEcp1-ISKpn14 (Figure 2C).

Mobile genetic element analysis revealed extensive IS26 distribution across the plasmids, particularly associated with resistance gene clusters. The dense presence of insertion sequences, transposons, and integrative elements surrounding ARGs demonstrated the dynamic genetic architecture facilitating horizontal gene transfer. This multi-plasmid resistance framework, encompassing three distinct replicon combinations with varying genetic environments, characterized the complex resistance dissemination mechanisms present in the ST101 isolates.

### 2.8. Phylogenetic Relationships of ST101 Isolates

**Figure 3:**
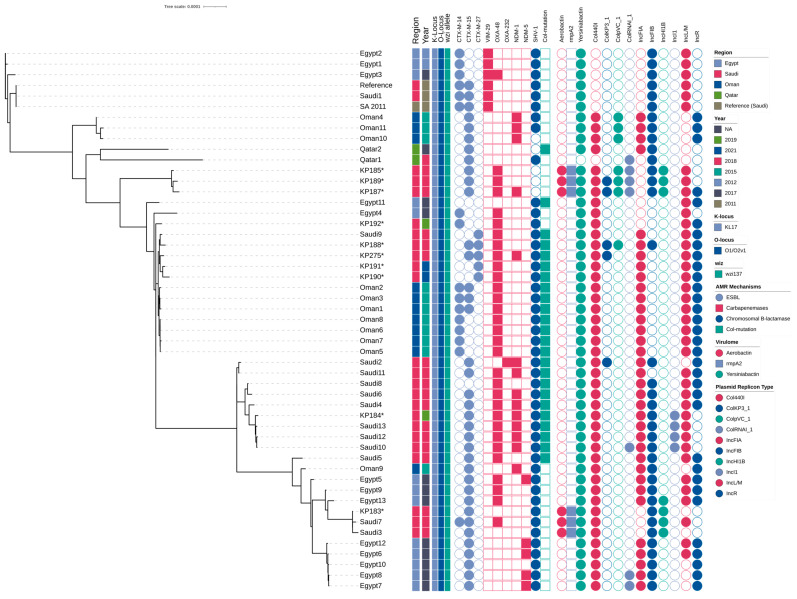
Phylogenetic Analysis and Resistance Profile of ST101 Isolates. Maximum-likelihood phylogenetic tree based on core genome single-nucleotide polymorphisms (SNPs) of *K. pneumoniae* isolates from Saudi Arabia and neighboring Gulf countries. Isolates from this study are indicated with asterisks (*). The adjacent heatmap displays the presence or absence of acquired extended-spectrum β-lactamases (ESBL), carbapenemases, chromosomal β-lactamases, and chromosomal colistin resistance mutations (*mgrB*).

Phylogenomic reconstruction was performed using maximum likelihood analysis implemented in IQ-TREE multicore (version 2.3.6) with automatic model selection, and the resulting tree was visualized using Interactive Tree Of Life (iTOL; version 7). The phylogenetic analysis revealed minimal genetic divergence among study isolates, with short branch lengths indicating recent evolutionary relationships and potential clonal expansion within the ST101 lineage. Genomic clustering analysis identified distinct epidemiological patterns within the study population. Isolate KP183 and several related strains formed a tight cluster, demonstrating high genetic similarity consistent with recent common ancestry or ongoing transmission networks. Phylogeographic analysis revealed that Saudi Arabian isolates clustered with Egyptian ST101 strains, indicating trans-regional dissemination patterns and shared evolutionary origins within the Middle Eastern population structure. A subset of Saudi Arabian isolates formed intermediate clades with other regional isolates, suggesting established circulation of ST101 variants within the Arabian Peninsula. These relationships indicate either ancestral diversification events or multiple independent introduction events followed by local adaptation and expansion. Notably, three isolates (KP190, KP191, KP275) constituted a highly supported a tight clade with minimal internal branch lengths, indicating recent clonal expansion or potential nosocomial transmission. This clustering pattern demonstrates the utility of whole-genome sequencing for high-resolution epidemiological surveillance and outbreak investigation of CRKP clones. Comparative phylogeographic analysis revealed contrasting population structures between regional populations. Egyptian isolates exhibited limited genetic diversity with tight clustering, consistent with clonal expansion from a recent common ancestor or founder effect. In contrast, Saudi Arabian isolates displayed greater phylogenetic diversity with distribution across multiple sub-lineages, indicating more complex introduction patterns or longer-term endemic circulation within the healthcare system (Figure 3).

## 3. Discussion

Carbapenem-resistant *Klebsiella pneumoniae* strains represent a critical global public health threat through their capacity for rapid horizontal dissemination of resistance determinants, resulting in difficult-to-treat infections with increased mortality rates and adverse clinical outcomes across healthcare systems worldwide [26]. Recent European surveillance studies have identified *K. pneumoniae* ST101 as an emerging high-risk clone characterized by widespread dissemination of carbapenem and colistin resistance mechanisms, accompanied by elevated morbidity and mortality rates [21,22,27,28]. In Saudi Arabia, *K. pneumoniae* ST101 has been recognized as one of the predominant MDR clones driving regional resistance dissemination [24]. This investigation presents comprehensive findings from a four-year CRKP surveillance program conducted at MNGHA hospitals throughout Saudi Arabia, during which ST101 emerged as one of the predominant circulating clones. Our analysis encompasses detailed clinical epidemiology alongside comprehensive phenotypic and genotypic characterization of collected isolates, with particular emphasis on plasmid architecture and mobile genetic element analysis. Furthermore, this study elucidates critical molecular mechanisms underlying resistance gene acquisition and dissemination within the ST101 clone of CRKP circulating throughout Saudi Arabian healthcare networks. While our analysis focused on ten ST101 isolates, which may not fully capture the complete genetic diversity of this lineage across the country, these findings provide essential insights into regional resistance patterns. Therefore, results should be interpreted within this context while recognizing their significant contribution to understanding ST101 epidemiology in the Middle Eastern region.

As part of a comprehensive surveillance initiative, CRKP ST101 isolates were recovered from patients across three major regions in Saudi Arabia Riyadh, Jeddah, and Al-Ahsa enabling comparative genomic analysis across distinct healthcare settings and geographic distributions. The temporal distribution revealed that the majority of isolates were collected during 2018, with no isolates obtained in 2021, likely reflecting reduced clinical sampling and operational constraints associated with COVID-19 pandemic disruptions. Clinical epidemiological analysis demonstrated that over 80% of isolates were obtained from hospitalized patients, indicating that CRKP ST101 infections are predominantly hospital-acquired rather than community-acquired [29]. This nosocomial predominance was further reinforced by the observation that all ten ST101 isolates analyzed were recovered from inpatient settings, emphasizing the healthcare-associated transmission patterns characteristic of this high-risk clone. Patient demographics revealed significant underlying comorbidities, including diabetes mellitus, hypertension, and dyslipidemia, which represent established risk factors for adverse outcomes in MDR bacterial infections. Clinical presentations included multiple patients admitted with critical conditions, and comprehensive infection classification identified all cases as healthcare-associated infections (HAIs), with no community-acquired infections documented throughout the surveillance period. Although clinical outcome data availability was limited, a substantial proportion of patients experienced severe adverse outcomes, including in-hospital mortality, with infection-related deaths documented in multiple cases. These clinical findings align with previous investigations reporting elevated morbidity and mortality rates associated with CRKP infections, particularly among vulnerable patient populations with underlying chronic disease conditions [21,27].

Comprehensive phenotypic susceptibility testing revealed extensive multidrug resistance across multiple antibiotic classes, including β-lactams, fluoroquinolones, and sulfonamides, reflecting the complex resistance architecture harbored by these ST101 isolates. This broad resistance profile substantially constrained available therapeutic options, with susceptibility retained primarily to colistin, tigecycline, and, in select cases, aminoglycosides, thereby limiting clinicians to last-resort antimicrobial agents. The therapeutic implications of this resistance pattern present significant clinical challenges, as CRKP ST101 infections often necessitate the use of last-resort antimicrobials associated with considerable toxicity profiles and variable clinical efficacy. Although plasmid-mediated colistin resistance among *K. pneumoniae* ST101 strains has been increasingly documented in global surveillance studies [27], none of our isolates carried plasmid-borne *mcr* genes. Instead, colistin resistance mechanisms in our cohort were attributed to chromosomal mutations within the *mgrB* regulatory gene, which was detected in (n = 5, 50%) of the analyzed isolates. This chromosomal resistance pattern demonstrates the critical role of mutation-driven mechanisms in mediating colistin resistance among CRKP within our healthcare setting.

Furthermore, these findings underscore the critical importance of differentiating plasmid-mediated from chromosomally encoded resistance when shaping antimicrobial-resistance surveillance frameworks and infection-control policies. The genomic context of resistance genes fundamentally determines their epidemiological behavior. Plasmid-borne determinants can disseminate rapidly across bacterial lineages and species through conjugative transfer, fueling interhospital and interregional outbreaks. In contrast, chromosomal integration of resistance genes ensures vertical inheritance and long-term stability within successful clonal lineages, often with a reduced fitness burden. These distinct evolutionary pathways profoundly influence diagnostic interpretation and containment strategies: plasmid-encoded ARGs signal active horizontal-gene-transfer events, whereas chromosomal integration represents entrenched resistance within established clones. Recognizing this dichotomy is essential for anticipating transmission dynamics and for designing precision genomic-surveillance systems that effectively mitigate the regional and global spread of AMR [30,31].

Comprehensive phenotypic-genotypic correlation analysis revealed that all carbapenemase-producing isolates were accurately identified through phenotypic susceptibility testing, demonstrating complete concordance with molecular detection methods and validating the reliability of laboratory diagnostic approaches. However, one isolate exhibited a carbapenem-resistant phenotype despite the absence of detectable carbapenemase-encoding genes, representing an important diagnostic consideration for clinical laboratories. This phenomenon is well-documented in the literature and typically results from alternative resistance mechanisms, including overexpression of extended-spectrum β-lactamases in combination with compromised outer membrane permeability [32,33]. Consistent with this established mechanism, molecular characterization revealed that the isolate harbored the *bla*_CTX-M-15_ gene alongside disruptive mutations in the ompK35 and ompK36 porin genes, which represent likely contributors to the observed carbapenem-resistant phenotype [34,35]. This finding emphasizes the complexity of carbapenem resistance mechanisms and highlights the importance of comprehensive molecular characterization for accurate resistance profiling, particularly in isolates where phenotypic and genotypic results appear discordant.

Comprehensive molecular epidemiological analysis revealed the regional complexity and dissemination patterns of CRKP ST101 lineages across Middle Eastern healthcare networks. Notably, two isolates within our study population harbored both *bla*_CTX-M-15_ and *bla*_CTX-M-27_ ESBL genes, representing a rare co-occurrence that suggests active plasmid-mediated genetic exchange and the emergence of highly resistant sub-lineages with enhanced therapeutic evasion potential. This dual ESBL gene acquisition pattern raises significant concerns regarding local adaptation mechanisms and the evolution of increasingly complex resistance architectures. Comparative genomic analysis utilizing the Pathogenwatch database demonstrated that our ST101 isolates exhibit close phylogenetic relationships with other strains circulating throughout the Middle Eastern region, indicating established regional clonal dissemination networks. These phylogenetically related clones demonstrate similar resistance patterns despite considerable variation in their plasmid content profiles, reflecting the dynamic acquisition and loss of plasmid-mediated resistance determinants within regional healthcare systems. Furthermore, several Egyptian isolates clustered closely with Saudi Arabian strains, particularly those harboring *bla*_NDM-5_, suggesting potential epidemiological linkages or shared regional selective pressures driving resistance evolution. The presence of NDM-5-producing strains in Egypt, a carbapenemase gene frequently associated with high-level carbapenem resistance and enhanced plasmid mobility, reflects broader regional resistance dynamics that may significantly influence future dissemination patterns across Middle Eastern healthcare settings [36,37].

*K. pneumoniae* ST101 is globally recognized as a high-risk clone strongly associated with the dissemination of *bla*_OXA-48_ carbapenemase genes across healthcare systems worldwide [21,38,39,40]. According to comprehensive surveillance conducted in Europe, the same high-risk lineages identified during 2013-14 (EuSCAPE) continued to circulate across European hospitals in 2019, including ST11, ST15, ST101, and ST258/512. Moreover, concerning epidemiological shifts in pathogen populations were observed, including increased acquisition of carbapenemase genes by MDR lineages such as ST147, ST307, and ST39 [28]. Consistent with our findings, recent surveillance studies from Saudi Arabia have similarly identified ST101 as one of the predominant circulating clones harboring *bla*_OXA-48_ carbapenemase genes [24,41]. In our cohort, 20% of CRKP ST101 isolates co-harbored *bla*_NDM-1_ and *bla*_OXA-48_ genes, indicating the emergence of dual-carbapenemase producers with enhanced resistance potential. The identification of co-producing *bla*_OXA-48_ and *bla*_NDM-1_ carbapenemase genes underscores the critical need for early molecular diagnostics to guide optimal antibiotic selection and prevent inappropriate empiric therapy. A parallel study from Spain similarly reported co-presence of *bla*_NDM-1_, *bla*_OXA-48_ and *bla*_CTX-M-15_ genes in ST101 clinical isolates, supporting the hypothesis that globally circulating high-risk clones with complex resistance architectures are increasingly present within our region [42]. These findings demonstrate the evolving complexity of resistance mechanisms in high-risk clones and underscore the urgent need for sustained genomic surveillance and targeted infection control measures. Understanding the molecular mechanisms underlying plasmid-mediated resistance can directly support antimicrobial-stewardship initiatives aimed at minimizing unnecessary carbapenem use and preserving the efficacy of remaining last-line treatment options, including tigecycline and colistin. These findings demonstrate how genomic data can inform evidence-based prescribing and strengthen infection-control practices within healthcare systems facing the growing challenge of CRKP.

Our genomic analysis revealed that *bla*_OXA-48_ and *bla*_NDM-1_ genes were located on distinct plasmid systems, suggesting independent acquisition events and potential for differential horizontal transfer. However, we acknowledge that conjugation or transformation assays were not performed to experimentally validate their mobility potential. Future investigations incorporating conjugation experiments are warranted to confirm the horizontal transfer capacity of these resistance determinants and their epidemiological significance. This genomic investigation establishes one of the first comprehensive molecular epidemiology datasets of CRKP ST101 in Saudi Arabia, offering crucial insight into the evolutionary mechanisms driving the persistence of this globally disseminated high-risk clone. The concurrent presence of *bla*_OXA-48_ and *bla*_NDM-1_ carbapenemase genes on IncL/M-type plasmids, together with virulence-associated elements such as ICEKp3, indicates an adaptive genomic process that strengthens bacterial fitness and survival under antimicrobial pressure. These findings not only underscore the regional emergence of MDR *K. pneumoniae* lineages but also contribute essential data to global AMR surveillance efforts from a region where genomic information remains limited. Plasmid and resistance gene structures were inferred from short-read Illumina assemblies, which may not fully resolve repetitive genomic regions; future work integrating long-read sequencing (PacBio or Oxford Nanopore technologies) will enable high-resolution mapping of mobile genetic elements and refine our understanding of resistance evolution in ST101. Importantly, this work supports the ongoing national efforts in Saudi Arabia to strengthen genomic surveillance and early detection of AMR, reinforcing the Kingdom’s contribution to global AMR preparedness and One Health initiatives.

## 4. Materials and Methods

### 4.1. Hospital Setting and Study Design

This retrospective cross-sectional study was part of the AMR Surveillance Program initiated by the MNGHA in Saudi Arabia in 2018, led by the Infectious Disease Research Department (IDRD) at King Abdullah International Medical Research Centre (KAIMRC). The MNGHA operates a network of hospitals and specialized medical centers located in various regions, including Riyadh, Jeddah, Al-Ahsa, Dammam, and Medina, under the Ministry of National Guard and the Saudi Arabian National Guard. These facilities, with a capacity of over 3000 beds, primarily serve National Guard employees and their families, offering care from primary health services to advanced tertiary care. All *K. pneumoniae* isolates and their VITEK reports were collected from MNGHA hospital microbiology laboratories across the country and sent to the IDRD at KAIMRC. There, species identification and the presence of ARGs were confirmed through multiplex PCR. Isolates that met the selection criteria were further analyzed using whole-genome sequencing and advanced bioinformatics tools for deeper genetic insights.

### 4.2. Bacterial Isolates and Phenotypic Testing

Between January 2018 and December 2021, a total of ten CRKP clinical isolates were gathered from various clinical sources, like blood, urine, respiratory samples, wound tips, tissue, and other clinical types. These strains were sent by MNGHA hospital microbiology labs throughout Saudi Arabia. The isolates were first identified using the VITEK II automated system (BioMerieux, Marcy-l’Etoile, France) and then further validated through multiplex PCR. Antimicrobial susceptibility testing was conducted using the Gram-negative bacteria card on the VITEK II system. These CRKP isolates were further examined to identify resistance mechanisms, virulence factors, and the associated mobile genetic elements through molecular characterization. All clinical data was pulled from the MNGHA clinical system. Data collection and research was authorized by the Ethics Committee of KAIMRC.

### 4.3. Bacterial Identification and Antimicrobial Susceptibility Testing (AST)

The antimicrobial susceptibility testing for CRKP isolates was done on 16 antibiotic agents, which included ampicillin (AMP), amoxicillin/clavulanic acid (AMC), piperacillin/tazobactam (PTZ), cefaclor (CEF), cefoxitin (FOX), ceftazidime (CAZ), ceftriaxone (CRO), cefepime (FEB), imipenem (IMP), meropenem (MEM), amikacin (AMK), gentamicin (GEN), ciprofloxacin (CIP), tigecycline (TGC), nitrofurantoin (NIT), and trimethoprim/sulfamethoxazole (TMP/SMX). These were tested using the VITEK II automated system (BioMerieux, Marcy-l’Etoile, France) along with the (AST-N291) GN identification card. The interpretation of results was done according to the Clinical and Laboratory Standards Institute (CLSI) recommendations [25].

### 4.4. Genotypic Screening of Carbapenemases

All *K. pneumoniae* isolates with reduced carbapenem susceptibility were screened for carbapenemase genes (*bla*_OXA-48_, *bla*_NDM_, *bla*_KPC_, *bla*_IMP_, and *bla*_VIM_) using a Multiplex-PCR assay [9]. The assay was optimized for specificity, and results were validated with control strains. Gel electrophoresis confirmed the presence of the targeted resistance genes. the primers used for this essay are listed in (Table 3).

### 4.5. Whole Genome Sequencing and Bioinformatics Analysis

Each *K. pneumoniae* isolate was obtained from a single colony sub-cultured from the primary clinical specimen to ensure clonal purity. High-quality genomic DNA was extracted and sequenced from this purified colony to confirm genomic uniformity and avoid sequencing a heterogeneous population. DNA quality and concentration were assessed using a Qubit fluorometer (Thermo Fisher Scientific, Waltham, MA, USA). Sequencing libraries were prepared following the manufacturer’s protocol and subjected to paired-end whole-genome sequencing on the Illumina MiSeq^®^ platform (Illumina, San Diego, CA, USA) at the King Abdullah International Medical Research Center (KAIMRC), Riyadh, Saudi Arabia. The quality of the raw sequencing data was checked with FastQC “https://github.com/s-andrews/FastQC, (accessed on 15 April 2023)”, and de novo assembly of high-quality reads was performed using SPAdes (version 1.0.9) “https://github.com/ablab/spades (accessed on 15 March 2023)” [43]. Assembly quality assessment and stats like genome size, GC content, and N50 were identified using QUAST software (version 5.3.0) “https://github.com/ablab/quast (accessed on 23 April 2025)”. Then, the genomic sequences were characterized and annotated using advanced bioinformatics pipelines. Sequence types were determined using MLST (version 2.9) “https://github.com/tseemann/mlst (accessed on 24 April 2025)” [44], while ARGs, virulence-associated genes, and plasmid replicon typing were identified by ABRicate (version 0.5) “https://github.com/tseemann/abricate (accessed on 24 April 2025)” using the pre-downloaded ResFinder [45], VFDB [46], and PlasmidFinder databases [47], respectively. Sequence types, ARGs, virulence factor genes, resistance and virulence scores, and capsule serotype (K) and O antigen (LPS) serotype predictions were validated using Kleborate (version 2.3.2) “https://github.com/klebgenomics/Kleborate/tree/main (accessed on 26 April 2025)” [48].

### 4.6. Plasmid Characterization

The annotation for ARGs, virulence factors, and mobile genetic elements was performed using Prokka (version 1.13) and Artemis Software (version 18.2.0) [49,50,51,52,53]. Mauve software (version 2.4.0) was used for assembly quality assessment and for aligning genomic and open reading frame sequences using *K. pneumoniae* strain KP4823, complete genome (accession numbers: CP082791.1-CP082795.1) as a reference; BRIG (version 0.95) software was used to generate plasmid circle figures [54]. The complete genome of characterized plasmid replicon types were used as a reference (IncL/M: accession number: CP071281.1); (IncHI1B/IncFIB: accession number: CP071280.1); and (IncFIA/IncR: accession number: LC807787) [55,56].

### 4.7. Phylogenetic Analysis

All *K. pneumoniae* ST101 strains isolated in Saudi Arabia were retrieved from Pathogenwatch genome database. The whole genome of *K. pneumoniae* (accession number: SAMD00055765) was obtained from the Pathogenwatch genome database to be used as a reference. All *K. pneumoniae* ST101 sequences from Middle Eastern countries available in the Pathogenwatch genome database were retrieved and incorporated into the phylogenetic analysis. The accession numbers and associated metadata for these sequences are provided in the supplementary file Table S1. Phylogenetic analysis of the isolates was conducted by identifying single-nucleotide polymorphisms (SNPs) using Snippy (version 4.6.0) “https://github.com/tseemann/snippy, (accessed on 9 May 2025)”. To ensure precise reconstruction of evolutionary relationships under realistic short-term bacterial evolution models, Gubbins (version 3.3.5) “https://github.com/nickjcroucher/gubbins, (accessed on 10 May 2025)” was employed [57]. The output from Gubbins was subsequently used to construct the phylogenetic tree with IQ-TREE (version 2.3.6) “https://github.com/iqtree/iqtree2, (accessed on 11 May 2025)” [58,59]. A treefile that was generated by IQ-TREE was annotated and visualized in the interactive Tree of Life (iTOL) (version 7.0) “https://itol.embl.de, (accessed on 16 May 2025)”.

### 4.8. Statistical Analysis

All data were presented as frequencies and percentages. Microsoft Excel was used for the quantitative analysis of the collected data and GraphPad Prism (version 10.2.3) (347) was used in creating the graphs and figures.

## 5. Conclusions

This investigation reveals *K. pneumoniae* ST101 as an evolutionary paradigm shift in AMR, representing the emergence of a “super-pathogen” that fundamentally threatens the foundation of modern medicine. Our comprehensive genomic architecture analysis demonstrates the unprecedented convergence of dual carbapenemases (*bla*_OXA-48_/*bla*_NDM-1_) with multiple extended-spectrum β-lactamases (*bla*_CTX-M-15_/*bla*_CTX-M-27_) within sophisticated multi-plasmid resistance platforms, creating an essentially pan-resistant phenotype that renders the entire antimicrobial armamentarium ineffective.

These findings represent a watershed moment in the global AMR crisis, marking the transition from treatable MDR infections to virtually untreatable pan-resistant pathogens. The complex molecular architecture we have elucidated, encompassing three distinct plasmid systems (IncL/M, IncHI1B/IncFIB, IncFIA/IncR) with extensive mobile genetic element landscapes, establishes a highly efficient horizontal gene transfer network capable of rapidly disseminating this resistance constellation across bacterial populations worldwide. The strategic emergence of these isolates within Saudi Arabia, positioned at the crossroads of global healthcare networks, amplifies their pandemic potential and represents an immediate existential threat to international health security.

Our molecular epidemiological analysis provides the critical foundation for paradigm-shifting public health interventions. The genomic signatures and resistance architectures we have characterized enable the development of next-generation rapid diagnostics, precision surveillance systems, and targeted containment strategies, which are essential for preventing global dissemination of this emerging super-pathogen. This work establishes *K. pneumoniae* ST101 as the highest priority target for enhanced global genomic surveillance and underscores the critical urgency for revolutionary therapeutic approaches, including novel antimicrobial classes and alternative treatment modalities, to combat what may represent the vanguard of a post-antibiotic era.

## Figures and Tables

**Table 1:** Clinical characteristics, outcomes, and comorbidities of patients infected with *K. pneumoniae* strains. This table presents detailed clinical data for patients infected with *K. pneumoniae*, including inpatient (IP) or outpatient (OP) status, hospital department, patient outcomes, reasons for admission, causes of death (where applicable), and presence of healthcare-associated infection (HAI) or community-acquired infection (CAI). It also documents age, comorbid conditions such as diabetes mellitus, hypertension, dyslipidemia, and other chronic illnesses (e.g., chronic kidney disease, dementia, hypothyroidism). High mortality, particularly among intensive care unit (ICU) patients, underscores the severe impact of these MDR infections.

Strains	Collection Department	Outcome	Admission Reason/Visit	Death Reason	Death During Admission	HAI	CAI	Death Due to Infection
KP183	ICU	Died	Mucormycosis status post FESS and debridement with excision of hard palate/ maxillary sinus	Severe pulmonary edema, septic shock	Yes	Yes	-	Yes
KP184	ICU	Died	Shortness of breath	Multiorgan failure, cardiac arrest	Yes	-	Yes	Yes
KP185	ICU	Died	Hemorrhagic stroke	Septic shock due to CRKP, multiorgan failure	Yes	Yes	-	Yes
KP187	ICU	Died	Stoma closure	Septic shock with multiorgan failure	Yes	Yes	-	Yes
KP188	IP	Improved	Shortness of breath	-	-	-	Yes	-
KP189	ICU	Died	Complete paralysis of his right leg	Septic shock	Yes	Yes	-	Yes
KP190	IP	Improved	gastrointestinal bleeding, melena	-	-	Yes	-	-
KP191	OP	Improved	Urinary Tract Infection	-	-	-	Yes	-
KP192	IP	NA	NA	NA	NA	NA	NA	NA
KP275	ICU	Died	Cardiac arrest	Fulminant sepsis, multiorgan failure, septic shock	Yes	Yes	-	Yes

**Table 2:** Genomic and phenotypic characteristics of CPKP carbapenemase-producing *K. pneumoniae* isolates from various regions in Saudi Arabia (2018–2021).

Strain	Year of Isolation	Region	Specimen Type	Plasmid-Associated Genes			Plasmid Replicon Typing	Chromosome-Associated Genes		Serotyping	Virulence Determinants		
				Carbapenemases	ESBL	Other Resistance Genes		ß-Lactamases	Colistin		Yersiniabactin	Aerobactin	*rmpA2*
KP183	2018	Jeddah	Respiratory	-	*bla*_CTX-M-15_	*aph3-Ia*; *arm*A; *mph*A; *mph*E; *msr*E; *sul*1; *sul*2; *dfr*A	Col440I, IncFIB, IncHI1B	*bla*_SHV-1_	-	KL17; O1/O2v1	*ybt9*; ICEKp3	*iuc*1	*rmpA2*_6-60%
KP184	2019	Jeddah	Respiratory	*bla*_NDM-1_; *bla*_OXA-48_	*bla*_CTX-M-15_	*aac*(6′)*-Ib*; *aad*A12; *aph*(3′)*-VI*; *qnr*S; *erm*B; *mph*A; *sul*1; *tet*(D), *dfr*A	Col440I, IncFIA, IncFIB, IncL/M, IncI1	*bla*_SHV-1_	*mgrB (altered)*	KL17; O1/O2v1	*ybt9*; ICEKp3	-	-
KP185	2018	Riyadh	Blood	*bla*_OXA-48_	*bla*_CTX-M-15_	*aac(3)-IId*; *aac*(6′)*-Ib*; *aad*A; *aph*(3′)*-VI*; *cat*A; *sul*2; *dfr*A	Col440I, ColpVC, ColRNAI, IncFIB, IncHI1B, IncL/M	-	-	KL17; O1/O2v1	*ybt9*; ICEKp3	*iuc*1	*rmpA2*_6-60%
KP187	2018	Riyadh	Respiratory	*bla*_NDM-1_; *bla*_OXA-48_	*bla*_CTX-M-15_	*aac(3)-IId*; *aac*(6′)*-Ib*; *aad*A; *aad*A2; *sat*-2; *str*B.v1; *cat*A; *sul*2; *dfr*A	Col440I, ColKP3, ColpVC, ColRNAI, IncFIB, IncHI1B, IncL/M, IncR	-	-	KL17; O1/O2v1	*ybt9*; ICEKp3	*iuc*1	*rmpA2*_6-60%
KP188	2018	Riyadh	Rectal swab	*bla*_OXA-48_	*bla*_CTX-M-15,_ *bla*_CTX-M-27_	*aac(3)-IIa*; *aac*(6′)*-Ib*; *aadA*2; *str*A; *mph*A; *cml*A; *sul*3; *dfr*A	Col440I; ColKP3, ColpVC, IncFIA, IncFIB, IncL/M, IncR	*bla*_SHV-1_	*mgrB (altered)*	KL17; O1/O2v1	*ybt9*; ICEKp3	-	-
KP189	2018	Riyadh	Blood	*bla*_OXA-48_	*bla*_CTX-M-15_	*aac(3)-IId*; *aac*(6′)*-Ib*; *aad*A; *aadA*2; *sat-2*; *strB.v1*; *mph*E; *cat*A; *sul*2; *dfr*A	Col440I, ColKP3; ColpVC; ColRNAI; IncFIB, IncHI1B, IncL/M	-	-	KL17; O1/O2v1	*ybt9*; ICEKp3	*iuc*1	*rmpA2*_6-60%
KP190	2021	Riyadh	Urine	*bla*_OXA-48_	*bla*_CTX-M-27_	*aac(3)-IIa*; *str*A; *mph*A; *dfr*A	Col440I, IncFIA, IncL/M, IncR	*bla*_SHV-1_	*mgrB (altered)*	KL17; O1/O2v1	*ybt9*; ICEKp3	-	-
KP191	2021	Riyadh	Urine	*bla*_OXA-48_	*bla*_CTX-M-27_	*aac(3)-IIa*; *str*A; *mph*A; *dfr*A	Col440I, IncFIA, IncL/M, IncR	*bla*_SHV-1_	*mgrB (altered)*	KL17; O1/O2v1	*ybt9*; ICEKp3	-	-
KP192	2019	Ahsa	Wound	*bla*_OXA-48_	*bla*_CTX-M-14_	*aac*(6′)*-Ib*; *aad*A; *aph*(3′)*-VI*; *str*A; *mph*A; *dfr*A	Col440I, ColKP3, IncFIA, IncL/M, IncR	*bla*_SHV-1_	-	KL17; O1/O2v1	*ybt9*; ICEKp3	-	-
KP275	2018	Riyadh	Blood	*bla*_NDM-1_; *bla*_OXA-48_	*bla*_CTX-M-15,_ *bla*_CTX-M-27_	*aac(3)-IIa*; *aac*(6′)*-Ib*; *aad*A; *str*A; *mph*A; *dfr*A	Col440I, IncL/M, IncR	*bla*_SHV-1_	*mgrB (altered)*	KL17; O1/O2v1	*ybt9*; ICEKp3	-	-

**Table 3:** Primers sequences of carbapenemase genes used in Multiplex-PCR assay.

Resistance Gene	Primer ^a^	Primer Sequence (5′-3′)
*bla*_OXA-48_	OXA-F OXA-R	GGTTAAGGATGAACACCAAGTC TTGTGATGGCTTGGCGCAG
*bla*_NDM_	NDM-F NDM-R	ACCGAATGTCTGGCAGCACA GGGCCGTATGAGTGATTGC
*bla*_KPC_	KPC-F KPC-R	TCTGCTGTCTTGTCTCTCATG CTTGTCATCCTTGTTAGGCG
*bla_IMP_*	IMP-F IMP-R	GGAATAGAGTGGCTTAAYTCTC GGTTTAAYAAAACAACCACC
*bla_VIM_*	VIM-F VIM-R	GATGGTGTTTGGTCGCATA CGAATGCGCAGCACCAG

^a^ Forward, sense primer; Reverse, antisense primer.

## Data Availability

The whole-genome sequences of the chromosome and plasmids of *K. pneumoniae* ST101 isolates generated in this study have been deposited in the NCBI GenBank database under BioProject accession number PRJNA1192313. Individual genome accessions are available under SAMN50015027–SAMN50015036.

## Supplementary Materials

The following supporting information can be downloaded at: https://www.mdpi.com/article/10.3390/ijms262311518/s1.
