# Supplementary material for: Genomic Characterization and Resistance Mechanisms of Carbapenem-Resistant Klebsiella pneumoniae ST101 Isolates from Saudi Arabia
Source: Int J Mol Sci. 2025 Nov 27;26(23):11518. doi: 10.3390/ijms262311518 (PMC12691828; doi:10.3390/ijms262311518)
Supplement: Supplementary file 1 [file ijms-26-11518-s001.zip › ijms-3945842-supplementary.pdf]

## Supplementary Materials

# Genomic Characterization and Resistance Mechanisms of Carbapenem-Resistant *Klebsiella pneumoniae* ST101 Isolates from Saudi Arabia

**Enaam K. Idrees [1,2], Manal M. Alkhulaifi [1], Marwh G. Aldriwesh [2,3,4], Nasser Alqurainy [2,4,5], Liliane Okdah [2], Abdulrahman A. Alswaji [2], Eisa T. Alrashidi [2], Alhanouf S. Alshahrani [2], Sameera M. Al Johani [2,4,5], MNGHA Surveillance Group [4], Hanan H. Balkhy [2] and Majed F. Alghoribi [2,4,5,*]**

[1] Department of Botany and Microbiology, College of Science, King Saud University, Riyadh 11451, Saudi Arabia
[2] Infectious Diseases Research Department, King Abdullah International Medical Research Center, Riyadh 11481, Saudi Arabia, Riyadh 11481, Saudi Arabia
[3] **Department of Clinical Laboratory Sciences, College of Applied Medical Sciences, King Saud bin Abdulaziz University for Health Sciences, Riyadh 11481, Saudi Arabia**
[4] Ministry of the National Guard-Health Affairs, Riyadh 11426, Saudi Arabia
[5] Department of Basic Science, College of Science and Health Professions, King Saud bin Abdulaziz University for Health Sciences, Riyadh 14611, Saudi Arabia
*Correspondence: alghoribima@gmail.com; Tel.: +966500415566

*** Corresponding author:**

E-mail: alghoribima@gmail.com

Mailing address: Infectious Diseases Research Department, King Abdullah International Medical Research Center, Riyadh 11481, Saudi Arabia

Phone: +966500415566

**Supplementary Table S1. Metadata and accession details of *K. pneumoniae* isolates used for phylogenetic analysis.**

This table lists the metadata of 39 *K. pneumoniae* isolates retrieved from Pathogenwatch, including accession numbers, year and country of collection, geographic coordinates (latitude and longitude), and accession identifiers for study, experiment, and sequencing runs. The table also details the isolation source (e.g., human body sites, screening, blood), host type (human), and whether the sample was collected from an environmental setting. These isolates were used to construct the phylogenetic tree and comparative genomic heatmaps.

| Code | Accession Number | Year of collection | Country | latitude | longitude | Study accession | Experiment accession | Run accession | Isolation source | Host | Environment |
|---|---|---|---|---|---|---|---|---|---|---|---|
| **Saudi1** | SAMD00055765 | 2011 | Saudi | 23.71667 | 44.11667 | PRJDB4948 | DRX059756 | DRR065611 | NA | Human | FALSE |
| **Saudi2** | SAMEA6531285 | 2018 | Saudi | 23.71667 | 44.11667 | PRJEB36683 | ERX3901258 | ERR3891022 | Human body sites or biosamples | Human | FALSE |
| **Saudi3** | SAMEA6531307 | 2018 | Saudi | 23.71667 | 44.11667 | PRJEB36683 | ERX3901280 | ERR3891044 | Human body sites or biosamples | Human | FALSE |
| **Saudi4** | SAMEA6531328 | 2018 | Saudi | 23.71667 | 44.11667 | PRJEB36683 | ERX3901301 | ERR3891065 | Human body sites or biosamples | Human | FALSE |
| **Saudi5** | SAMEA6531355 | 2018 | Saudi | 23.71667 | 44.11667 | PRJEB36683 | ERX3901328 | ERR3891092 | Human body sites or biosamples | Human | FALSE |
| **Saudi6** | SAMEA6531370 | 2018 | Saudi | 23.71667 | 44.11667 | PRJEB36683 | ERX3901343 | ERR3891107 | Human body sites or biosamples | Human | FALSE |
| **Saudi7** | SAMEA6531380 | 2018 | Saudi | 23.71667 | 44.11667 | PRJEB36683 | ERX3901353 | ERR3891117 | Human body sites or biosamples | Human | FALSE |
| **Saudi8** | SAMEA6531406 | 2018 | Saudi | 23.71667 | 44.11667 | PRJEB36683 | ERX3901379 | ERR3891143 | Human body sites or biosamples | Human | FALSE |
| **Saudi9** | SAMEA6531455 | 2018 | Saudi | 23.71667 | 44.11667 | PRJEB36683 | ERX3901427 | ERR3891191 | Human body sites or biosamples | Human | FALSE |
| **Saudi10** | SAMEA6531461 | 2018 | Saudi | 23.71667 | 44.11667 | PRJEB36683 | ERX3901433 | ERR3891197 | Human body sites or biosamples | Human | FALSE |
| **Saudi11** | SAMEA6531474 | 2018 | Saudi | 23.71667 | 44.11667 | PRJEB36683 | ERX3901446 | ERR3891210 | Human body sites or biosamples | Human | FALSE |
| **Saudi12** | SAMEA6531484 | 2018 | Saudi | 23.71667 | 44.11667 | PRJEB36683 | ERX3901456 | ERR3891220 | Human body sites or biosamples | Human | FALSE |
| **Saudi13** | SAMEA6531487 | 2018 | Saudi | 23.71667 | 44.11667 | PRJEB36683 | ERX3901459 | ERR3891223 | Human body sites or biosamples | Human | FALSE |
| **Egypt1** | SAMN10141025 | 2012 | Egypt | 30.0444 | 31.2357 | PRJNA493667 | SRX4747174 | SRR7912005 | NA | Human | FALSE |

| | | | | | | | | | | | |
|---|---|---|---|---|---|---|---|---|---|---|---|
| **Egypt2** | SAMN10141026 | 2012 | Egypt | 30.0444 | 31.2357 | PRJNA493667 | SRX4747173 | SRR7912006 | NA | Human | FALSE |
| **Egypt3** | SAMN10527289 | NA | Egypt | 26 | 29 | PRJNA508509 | SRX5106455 | SRR8291867 | Urine | Human | FALSE |
| **Egypt4** | SAMN10527290 | NA | Egypt | 26 | 29 | PRJNA508509 | SRX5106460 | SRR8291872 | Urine | Human | FALSE |

| Code | Accession Number | Year of collection | Country | latitude | longitude | Study accession | Experiment accession | Run accession | Isolation source | Host | Environment |
|---|---|---|---|---|---|---|---|---|---|---|---|
| **Egypt5** | SAMN11388457 | NA | Egypt | 30.7865 | 31.0004 | PRJNA508510 | SRX5671325 | SRR8885598 | NA | Human | FALSE |
| **Egypt6** | SAMN11388458 | NA | Egypt | 30.7865 | 31.0004 | PRJNA508510 | SRX5671323 | SRR8885596 | NA | Human | FALSE |
| **Egypt7** | SAMN11388459 | NA | Egypt | 30.7865 | 31.0004 | PRJNA508510 | SRX5671328 | SRR8885601 | NA | Human | FALSE |
| **Egypt8** | SAMN11388460 | NA | Egypt | 30.7865 | 31.0004 | PRJNA508510 | SRX5671324 | SRR8885597 | NA | Human | FALSE |
| **Egypt9** | SAMN11388464 | NA | Egypt | 30.7865 | 31.0004 | PRJNA508510 | SRX5671332 | SRR8885605 | NA | Human | FALSE |
| **Egypt10** | SAMN11388466 | NA | Egypt | 30.7865 | 31.0004 | PRJNA508510 | SRX5671335 | SRR8885608 | NA | Human | FALSE |
| **Egypt11** | SAMN11388467 | NA | Egypt | 30.7865 | 31.0004 | PRJNA508510 | SRX5671331 | SRR8885604 | NA | Human | FALSE |
| **Egypt12** | SAMN11388484 | NA | Egypt | 30.7865 | 31.0004 | PRJNA508510 | SRX5671356 | SRR8885629 | NA | Human | FALSE |
| **Egypt13** | SAMN11388486 | NA | Egypt | 30.7865 | 31.0004 | PRJNA508510 | SRX5671363 | SRR8885636 | NA | Human | FALSE |
| **Oman1** | SAMN11853648 | 2015 | Oman | 21.4735 | 55.9754 | PRJNA544438 | SRX5896663 | SRR9122686 | Wound | Human | FALSE |
| **Oman2** | SAMN11853650 | 2015 | Oman | 21.4735 | 55.9754 | PRJNA544438 | SRX5896665 | SRR9122684 | Wound | Human | FALSE |
| **Oman3** | SAMN11853651 | 2015 | Oman | 21.4735 | 55.9754 | PRJNA544438 | SRX5896668 | SRR9122681 | Fecal screening | Human | FALSE |
| **Oman4** | SAMN13041994 | 2015 | Oman | 21.4735 | 55.9754 | PRJNA544438 | SRX7007817 | SRR10294951 | Wound | Human | FALSE |
| **Oman5** | SAMN13042009 | 2015 | Oman | 21.4735 | 55.9754 | PRJNA544438 | SRX7007833 | SRR10294935 | Wound | Human | FALSE |
| **Oman6** | SAMN13042028 | 2015 | Oman | 21.4735 | 55.9754 | PRJNA544438 | SRX7007854 | SRR10294914 | Wound | Human | FALSE |
| **Oman7** | SAMN13042038 | 2015 | Oman | 21.4735 | 55.9754 | PRJNA544438 | SRX7007865 | SRR10294903 | Catheter tip | Human | FALSE |
| **Oman8** | SAMN13042040 | 2015 | Oman | 21.4735 | 55.9754 | PRJNA544438 | SRX7007868 | SRR10294900 | Blood | Human | FALSE |
| **Oman9** | SAMN13042057 | 2015 | Oman | 21.4735 | 55.9754 | PRJNA544438 | SRX7007886 | SRR10294882 | Urine | Human | FALSE |
| **Oman10** | SAMN13042111 | 2015 | Oman | 21.4735 | 55.9754 | PRJNA544438 | SRX7007745 | SRR10295023 | Screening | Human | FALSE |

| | | | | | | | | | | | |
|---|---|---|---|---|---|---|---|---|---|---|---|
| **Oman11** | SAMN13042163 | 2015 | Oman | 21.4735 | 55.9754 | PRJNA544438 | SRX7007802 | SRR10294966 | Screening | Human | FALSE |
| **Qatar1** | SAMN13829675 | 2018 | Qatar | 25.28 | 51.53 | PRJNA599387 | SRX7874532 | SRR11267917 | Rectal swab | Human | FALSE |
| **Qatar2** | SAMN15813821 | 2017 | Qatar | 25.28 | 51.53 | PRJNA656934 | SRX9058015 | SRR12570031 | respiratory | Human | FALSE |
